# Identification of common molecular signatures of SARS-CoV-2 infection and its influence on acute kidney injury and chronic kidney disease

**DOI:** 10.3389/fimmu.2023.961642

**Published:** 2023-03-21

**Authors:** Weiwei Zhang, Leping Liu, Xiangcheng Xiao, Hongshan Zhou, Zhangzhe Peng, Wei Wang, Ling Huang, Yanyun Xie, Hui Xu, Lijian Tao, Wannian Nie, Xiangning Yuan, Fang Liu, Qiongjing Yuan

**Affiliations:** ^1^ Department of Nephrology, Xiangya Hospital of Central South University, Changsha, China; ^2^ Department of Pediatrics, The Third Xiangya Hospital of Central South University, Changsha, China; ^3^ Organ Fibrosis Key Lab of Hunan Province, Central South University, Changsha, China; ^4^ Health Management Center, Xiangya Hospital of Central South University, Changsha, China; ^5^ National Clinical Medical Research Center for Geriatric Diseases, Xiangya Hospital of Central South University, Changsha, China; ^6^ Research Center for Medical Metabolomics, Xiangya Hospital of Central South University, Changsha, China

**Keywords:** SARS-CoV-2, acute kidney injury, chronic kidney disease, differentially expressed genes, gene ontology, protein-protein interaction, hub gene, drug molecule

## Abstract

Severe acute respiratory syndrome coronavirus 2 (SARS-CoV-2) is the main cause of COVID-19, causing hundreds of millions of confirmed cases and more than 18.2 million deaths worldwide. Acute kidney injury (AKI) is a common complication of COVID-19 that leads to an increase in mortality, especially in intensive care unit (ICU) settings, and chronic kidney disease (CKD) is a high risk factor for COVID-19 and its related mortality. However, the underlying molecular mechanisms among AKI, CKD, and COVID-19 are unclear. Therefore, transcriptome analysis was performed to examine common pathways and molecular biomarkers for AKI, CKD, and COVID-19 in an attempt to understand the association of SARS-CoV-2 infection with AKI and CKD. Three RNA-seq datasets (GSE147507, GSE1563, and GSE66494) from the GEO database were used to detect differentially expressed genes (DEGs) for COVID-19 with AKI and CKD to search for shared pathways and candidate targets. A total of 17 common DEGs were confirmed, and their biological functions and signaling pathways were characterized by enrichment analysis. MAPK signaling, the structural pathway of interleukin 1 (IL-1), and the Toll-like receptor pathway appear to be involved in the occurrence of these diseases. Hub genes identified from the protein–protein interaction (PPI) network, including DUSP6, BHLHE40, RASGRP1, and TAB2, are potential therapeutic targets in COVID-19 with AKI and CKD. Common genes and pathways may play pathogenic roles in these three diseases mainly through the activation of immune inflammation. Networks of transcription factor (TF)–gene, miRNA–gene, and gene–disease interactions from the datasets were also constructed, and key gene regulators influencing the progression of these three diseases were further identified among the DEGs. Moreover, new drug targets were predicted based on these common DEGs, and molecular docking and molecular dynamics (MD) simulations were performed. Finally, a diagnostic model of COVID-19 was established based on these common DEGs. Taken together, the molecular and signaling pathways identified in this study may be related to the mechanisms by which SARS-CoV-2 infection affects renal function. These findings are significant for the effective treatment of COVID-19 in patients with kidney diseases.

## 1 Introduction

Severe acute respiratory syndrome coronavirus 2 (SARS-CoV-2) is a novel coronavirus that belongs to the *Coronaviridae* family and *Pisoniviricetes* class. SARS-CoV-2 has been found to cause severe respiratory problems when infecting the respiratory tract and is the main cause of COVID-19 (1, 2), which was estimated to have resulted in 18.2 million deaths worldwide during the pandemic in 2020 and 2021 (3). COVID-19 was initially deemed a febrile respiratory disease, but increasing evidence suggests that it is a complex multisystem disease (4, 5). Indeed, COVID-19 patients often exhibit manifestations of renal involvement in addition to respiratory symptoms (6). Acute kidney injury (AKI) is a common complication of COVID-19 that increases mortality, especially in intensive care unit (ICU) settings. Patients with chronic kidney disease (CKD) have a high risk of SARS-CoV-2 infection and COVID-19-related mortality (7–9).

AKI is the second most common complication in critically ill COVID-19 patients and is characterized by elevated serum creatinine, renal inflammation, and tubular necrosis. Epidemiologically, the incidence of AKI in COVID-19 patients is variable and depends on the severity of COVID-19, ranging from 10.5% to 37% (10). The pathophysiology of COVID-19-associated AKI is complex, and an increasing number of studies suggest that factors such as systemic inflammation and immune responses, activation of coagulation pathways, the renin–angiotensin system, and endothelial injury are involved in the process of renal damage that occurs in COVID-19 (9, 11, 12). Early reports indicated underlying CKD as a risk factor for COVID-19 severity and mortality (8, 9). The largest study included data from 17 million electronic health records and identified CKD as a risk factor for mortality in COVID-19 patients, with glomerular filtration rate (GFR)<30 ml/min/1.73 m^2^ and organ transplantation conferring a high risk in multivariate analyses (13). Additionally, a nationwide study in a US dialysis center reported higher seroprevalence of SARS-CoV-2 antibodies than in the general US population (14). AKI and CKD are often considered two separate stages of the same disease class (15, 16). Although most COVID-19 patients have improved renal function at discharge, the complex renal damage mechanisms of COVID-19 and the use of nephrotoxic drugs and mechanical ventilation during hospitalization suggest that further investigation is required to determine the long-term prognosis of renal function in COVID-19 patients (17–19).

The exact mechanism of SARS-CoV-2-related renal damage is not known. The main binding site for SARS-CoV-2, i.e., angiotensin-converting enzyme 2 (ACE2), is expressed at much higher levels in the kidney than in the lung (20–22). ACE2 is expressed apically in primary human airway epithelia (23), and previous studies have demonstrated that in COVID-19, pneumonia occurs as ACE2 levels increase in the cell membrane. In connection with a viral infection, the density level of ACE2 is extremely progressive in the lungs (24). Single-cell RNA sequencing analysis indicated that ACE2 is mainly expressed by glomerular parietal epithelial cells and proximal tubular cells. Other studies have suggested that SARS-CoV-2 can directly invade human kidney organoids through the ACE2 receptor (25). The infectivity of cells depends on not only ACE2 expression but also the types of proteases expressed. The cellular components required for virus entry into the kidney, such as cellular cathepsin L (CTSL) and transmembrane serine protease 2 (TMPRSS2), are also highly expressed, suggesting favorable conditions for the presence of SARS-CoV-2 in the kidneys (26). In addition, SARS-CoV-2 contributes to an imbalance in the renin–angiotensin–aldosterone system (RAAS) *via* ACE2, which may also exert deleterious hemodynamic effects involved in lung and kidney injury (27). Moreover, SARS-CoV-2 may target lymphocytes because they express ACE2, leading to lymphocyte activation, which consequently results in lymphocyte death and decreased immune protection (28). In patients with CKD, especially those with diabetic kidney disease (DKD), baseline downregulation of ACE2 and upregulation of ACE, a combination of proinflammatory and profibrotic states in the kidneys, might lead to CKD progression (11, 29). Therefore, the human kidney is a main target for SARS-CoV-2 infection, and it is necessary for researchers to further explore the complicated interactions between SARS-CoV-2 infection, AKI, and CKD.

**Figure 1:**
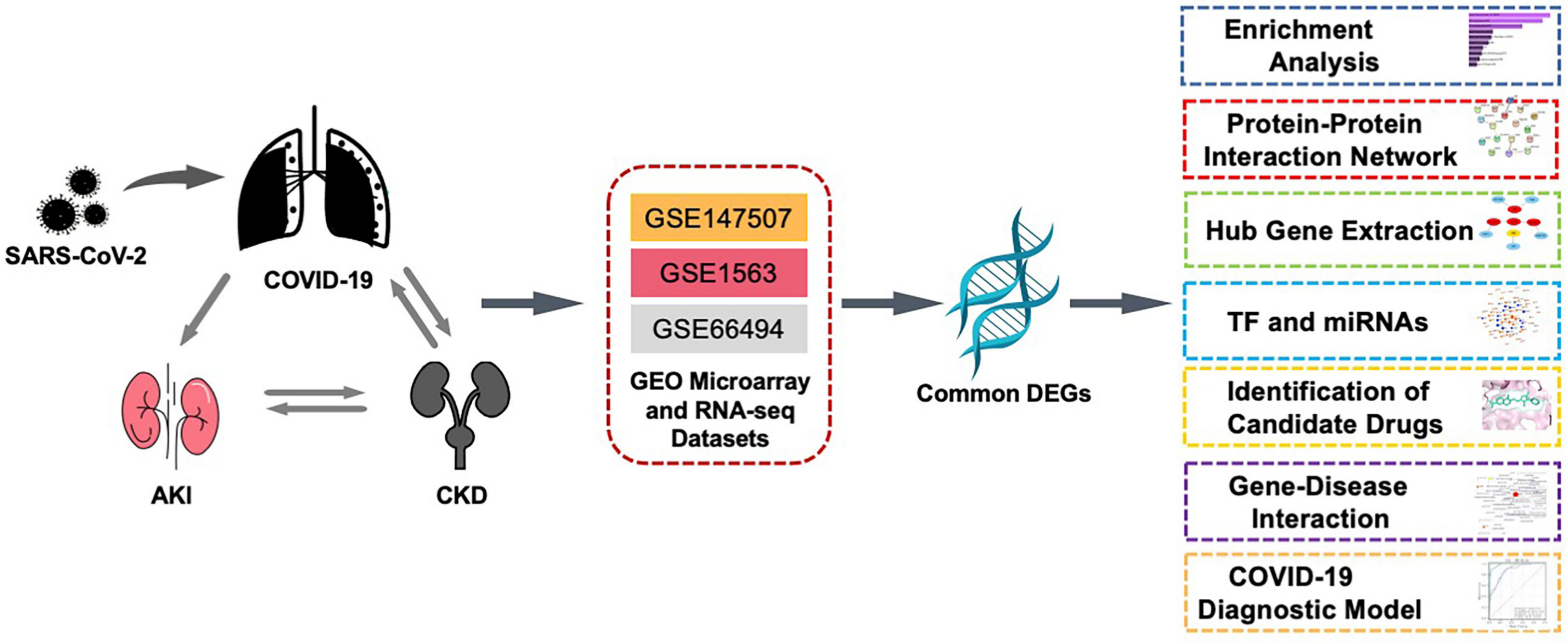
This diagram illustrates the overall workflow of the study. The author first found the common differentially expressed genes of COVID-19, AKI, and CKD and then analyzed the enriched functions, pathways, PPI networks, transcription factors and miRNAs, related diseases, and potential drugs of these differential genes. The three datasets, GSE1563, GSE66494, and GSE147507, in the figure represent the datasets of AKI, CKD, and COVID-19, respectively. AKI, acute kidney injury; CKD, chronic kidney disease; PPI, protein–protein interaction.

In this study, three datasets were used to explore the biological relationship between COVID-19, AKI, and CKD. These datasets were collected from the Gene Expression Omnibus (GEO) database, with GSE147507, GSE1563, and GSE66494 being used for COVID-19, AKI, and CKD, respectively. First, differentially expressed genes (DEGs) were confirmed using these datasets, and then common DEGs for the three diseases were identified and served as the main experimental genes for the entire study. These common DEGs were utilized for further experiments and analyses, including pathway and enrichment analyses, to understand the biological processes of genome expression studies. Extracting hub genes from common DEGs is essential for potential drug prediction, and a network of protein–protein interactions (PPIs) was also constructed *via* common DEGs to collect hub genes. Transcriptional regulators were also explored based on the common DEGs of GSE147507, GSE1563, and GSE66494, and potential drugs are suggested (**Figure 1**).

## 2 Materials and methods

### 2.1 Datasets employed in this study

To identify common genetic interactions among SARS-CoV-2, AKI, and CKD, microarray, and RNA-seq data were obtained from the National Center for Biotechnology Information (NCBI) (https://www.ncbi.nlm.nih.gov/geo/) GEO database (30). The SARS-CoV-2 dataset (GEO accession ID: GSE147507) involves transcriptional analysis of COVID-19 lung biopsies for respiratory infections using the Illumina NextSeq 500 platform for high-throughput sequencing. The AKI dataset (GEO accession ID: GSE1563) comprises human kidney tissue containing nine normal renal tissue samples and five AKI renal samples (samples from transplant patients with renal dysfunction without rejection), which were sequenced by Affymetrix Human Genome U95 Version 2 Array (31). The CKD dataset (GEO accession: ID GSE66494) was obtained from eight subjects with normal renal function and 54 CKD subjects (32); Agilent-014850 Whole Human Genome Microarray 4x44K G4112F was used to measure gene expression.

### 2.2 Identification of DEGs and mutual DEGs among AKI, CKD, and COVID-19

Genes are defined as distinctively expressed when statistically significant differences exist between the different levels of transcripts tested (33). DEGs for the acquired datasets GSE147507, GSE1563, and GSE66494 were first identified from long expression values with the LIMMA package and Benjamini–Hochberg calibration to control for the false discovery rate and DESEq2 in the R programming language (v 4.0.2) for multiple test options. Significant DEGs in the dataset were detected by cutoff criteria (p-value<0.05 and |logFC| ≥ 1.0), and mutual DEGs were obtained for GSE147507, GSE1563, and GSE66494 by the online VENN analysis tool Jvenn.

### 2.3 Gene Ontology and pathway enrichment analyses

The purpose of gene set enrichment analysis is to identify common biological insights, such as biological processes or chromosomal locations related to different diseases (34). Gene Ontology, functional enrichment, and pathway enrichment studies were performed with EnrichR (https://maayanlab.cloud/Enrichr/) to characterize the biological mechanisms and signaling pathways of the shared DEGs. Kyoto Encyclopedia of Genes and Genomes (KEGG), WikiPathways, and BioCarta were also used to identify shared pathways between AKI-, CKD-, and COVID-19-related metabolic processes. The top pathways were selected based on p-value<0.05.

### 2.4 Protein–protein interaction network analysis

AKI, CKD, and COVID-19 functional and physiological interactions were mapped with STRING (https://string-db.org/) (version 11.0). PPIs were examined using channels such as text mining, experimental databases, coexpression, culture, gene fusion, and co-occurrence under different settings of classification confidence scores (low, medium, and high) (35). Then, a medium confidence score of 0.5 was set to generate PPI networks for common DEGs. Cytoscape (v.3.7.1) was used to visualize PPIs, genetic interactions, protein–DNA interactions, and other types of interactions (36).

### 2.5 Hub gene extraction and submodule analysis

Nodes, edges, and connections exist between PPI networks, and nodes with high levels of cross-linking can be considered hub genes. CytoHubba (http://apps.cytoscape.org/apps/cytohubba) is a Cytoscape plug-in for ranking and extracting the key or potential targeted elements of biological networks based on various network characteristics. There are 11 methods for studying networks from different perspectives in cytoHubba, among which maximal clique centrality (MCC) is the best (37). By using the MCC method, the top 15 central genes were identified from the PPI network. The shortest possible paths between central genes were classified according to the closest neighboring feature of cytoHubba.

### 2.6 Recognition of TFs and miRNAs interacting with common DEGs

A transcription factor (TF) is a protein that binds to specific genes and controls the rate at which genetic information is transcribed. Therefore, TFs are crucial for molecular profiling. Topologically plausible TFs that tend to bind to our common DEGs were identified in the JASPAR database *via* the NetworkAnalyst platform. JASPAR is an openly available resource that collects profiles of TFs for numerous species in six taxonomic groups (38). NetworkAnalyst is an online platform for meta-analyzing gene expression data and obtaining insight into biological mechanisms, roles, and explanations. Furthermore, miRNAs targeting gene interactions are included to track the detrimental effects of miRNAs that target gene transcripts to affect protein expression (39). Both TarBase and mirTarBase are experimental validity databases for miRNA–target gene interactions (39, 40). MiRNAs interacting with common DEGs were obtained from TarBase and miRTarBase through miRNA–gene interactions from NetworkAnalyst. Topological analysis was performed by Cytoscape, and TF–gene and miRNA–gene interaction networks were identified. Using this tool, researchers can screen miRNAs with high rankings and detect biological functions and features to develop valid biological hypotheses.

### 2.7 Evaluation of applicant drugs and molecular docking

Through Enrichr, drug molecules were identified using the drug signature database (DSigDB) in relation to COVID-19, AKI, and CKD. Enrichr is a popular portal with a large number of different gene set libraries for exploring genome-wide enrichment of gene sets (41). DSigDB is a global archive for the identification of targeted drugs associated with DEGs (42). DSigDB, which contains 22,527 gene sets, can be accessed *via* Enrichr.

After drugs for the common DEGs were predicted by Enrichr, we downloaded the MOL2 form of these drugs from the ZINC (https://zinc.docking.org/) database (due to the lack of the MOL2 form of dimethyloxalylglycine in ZINC, we downloaded its SDF format from PubChem (https://pubchem.ncbi.nlm.nih.gov/)). We used openBabel software to convert the MOL and SDF formats of these small molecules to PDB formats. We downloaded the PDB format of DUSP6, BHLHE40, RASGRP1, TAB2, ACE2 (the functional host receptor of SARS-CoV-2), and 3CLpro (an enzyme necessary for SARS-CoV-2 replication) from Protein Data Bank (https://www.rcsb.org/). We used Autodock tools (version 1.5.4) to dock eight drugs and three proteins and then visualized the results with PyMOL Molecular Visualization System 2020 (PyMOL).

### 2.8 Molecular dynamics simulation

Based on the docking results for each protein and drug molecule, the drug–protein complex with the lowest binding energy was used as the initial structure for all-atom molecular dynamics simulations, and the simulation was performed using AMBER 18 software. Before the simulation, charges of the small molecules are calculated by the Hartree-Fock (HF) SCF/6-31G* of the antechamber module and Gaussian 09 software. Afterward, drug molecules and proteins are described using the GAFF2 small molecule force field and ff14SB protein force field, respectively. Each system utilizes the LEaP module to add hydrogen atoms to the system, add a truncated octahedral TIP3P solvent box at a distance of 10 Å in the system, add Na^+^/Cl^−^ to the system to balance the charge of the system, and output the topology and parameter file.

Molecular dynamics simulations were performed using AMBER 18 software. Before simulations, energy optimization of the system was carried out, including the steepest descent method with 2,500 steps and the conjugate gradient method with 2,500 steps. After the system energy optimization was completed, the temperature of the system was raised slowly from 0 to 298.15 K by 200 ps at a fixed volume and a constant heating rate. Under the condition that the system maintained a temperature of 298.15 K, a 500-ps NVT (isothermal isotropic) system simulation was performed such that the solvent molecules were further uniformly distributed in the solvent box. In the case of NPT (isothermal and isobaric), a 500-ps equilibrium simulation of the entire system was performed. Finally, under periodic boundary conditions, the two composite systems were simulated by 4-ns NPT (isothermal and isobaric) systems. During the simulation, the non-bond cutoff distance was set to 10 Å, the particle mesh Ewald (PME) method was used to calculate the long-range electrostatic interaction, the SHAKE method was applied to limit the length of hydrogen atomic bonds, and the Langevin algorithm (43) was used for temperature control, where the collision frequency γ was set to 2 ps-1. The system pressure is 1 atm, the integration step is 2 fs, and the trajectory is saved every 10 ps for subsequent binding energy calculations.

### 2.9 MM/GBSA binding free energy calculation

The free energies of binding between proteins and ligands in all systems were calculated by the molecular mechanics generalized Born surface area (MM/GBSA) method. In this study, the above molecular dynamics (MD) trajectory was used for calculation, and the specific formula is as follows:


$$\Delta G_{\text{bind}}=\Delta G_{\text{complex}}-(\Delta G_{\text{receptor}}+\Delta G_{\text{ligand}}),$$

$$=\Delta {\text{E}}_{\text{internal}}+\Delta {\text{E}}_{\text{VDW}}+\Delta {\text{E}}_{\text{elec}}+\Delta {\text{G}}_{\text{GB}}+\Delta {\text{G}}_{\text{SA}}$$

where ΔE_internal_ represents the internal energy, ΔE_VDW_ represents the van der Waals interaction, and ΔE_elec_ represents the electrostatic interaction. The internal energy includes the bond energy (E_bond_), angular energy (E_angle_), and torsion energy (E_torsion_); ΔG_GB_ and ΔG_SA_ are collectively referred to as solvation-free energy. Among them, G_GB_ is the free energy of polar solvation, and G_SA_ is the free energy of non-polar solvation. For ΔG_GB_, we used the GB model developed by researchers such as Nguyen (44) for calculation (*igb* = 2). The non-polar solvation free energy (ΔG_SA_) was calculated based on the surface tension (γ) multiplied by the solvent accessible surface area (surface area, SA), ΔG_SA_= 0.0072 × ΔSASA. Entropy change was neglected in this study due to high computational resource consumption and low precision.

### 2.10 Gene–disease association analysis

The DisGeNET project is a centralized database of gene–disease interactions obtained from a variety of sources and features various biomedical aspects of diseases. It highlights novel views of human genetic disorders (45). The network-analyst program was used to study gene–disease associations to discover the relationship between related diseases and chronic complications for the shared DEGs.

### 2.11 Construction of the COVID-19 diagnostic model

We used GSE147507 expression matrix information to establish a COVID-19 diagnosis model with fivefold cross-validation. We set 17 common DEGs as model key variables. Six different machine learning algorithms (“extreme gradient boosting (XGBoost)”, “light gradient boosting (LGBM)”, “RandomForest”, “Adaboost”, “support vector machine (SVC)”, and “k-nearest neighbor (KNN)”) were employed for modeling. The performance of each model was compared by a multimodel calibration curve and the area under the curve (AUC), and the best model was selected. After filtering out the best-performing models, we used the “SHapley Additive exPlanations (SHAP)” package in Python to explain the importance of key variables to the model and the contribution of each variable.

### 2.12 Statistical analysis

DEGs for three GEO datasets were first identified from long expression values with the LIMMA package and Benjamini–Hochberg calibration to control for the false discovery rate and DESEq2 in the R programming language (v 4.0.2) for multiple test options. Significant DEGs in the dataset were detected by cutoff criteria (p-value<0.05 and |logFC| ≥ 1.0).

Python software (version 3.7) was used to build the COVID-19 diagnostic model. During the modeling of various machine learning algorithms, the xgboost 1.2.1 package was applied to run the XGBoost algorithm, the lightgbm 3.2.1 package to run the LightGBM algorithm, and the sklearn 0.22.1 package to run other machine learning algorithms. The shap 0.39.0 package was used to demonstrate model interpretability. All statistical analyses in constructing the COVID-19 diagnostic model were carried out with Python version 3.7 and the Extreme Smart Analysis platform (https://www.xsmartanalysis.com/).

## 3 Results

### 3.1 Identification of DEGs and common DEGs among COVID-19, AKI, and CKD

**Figure 2:**
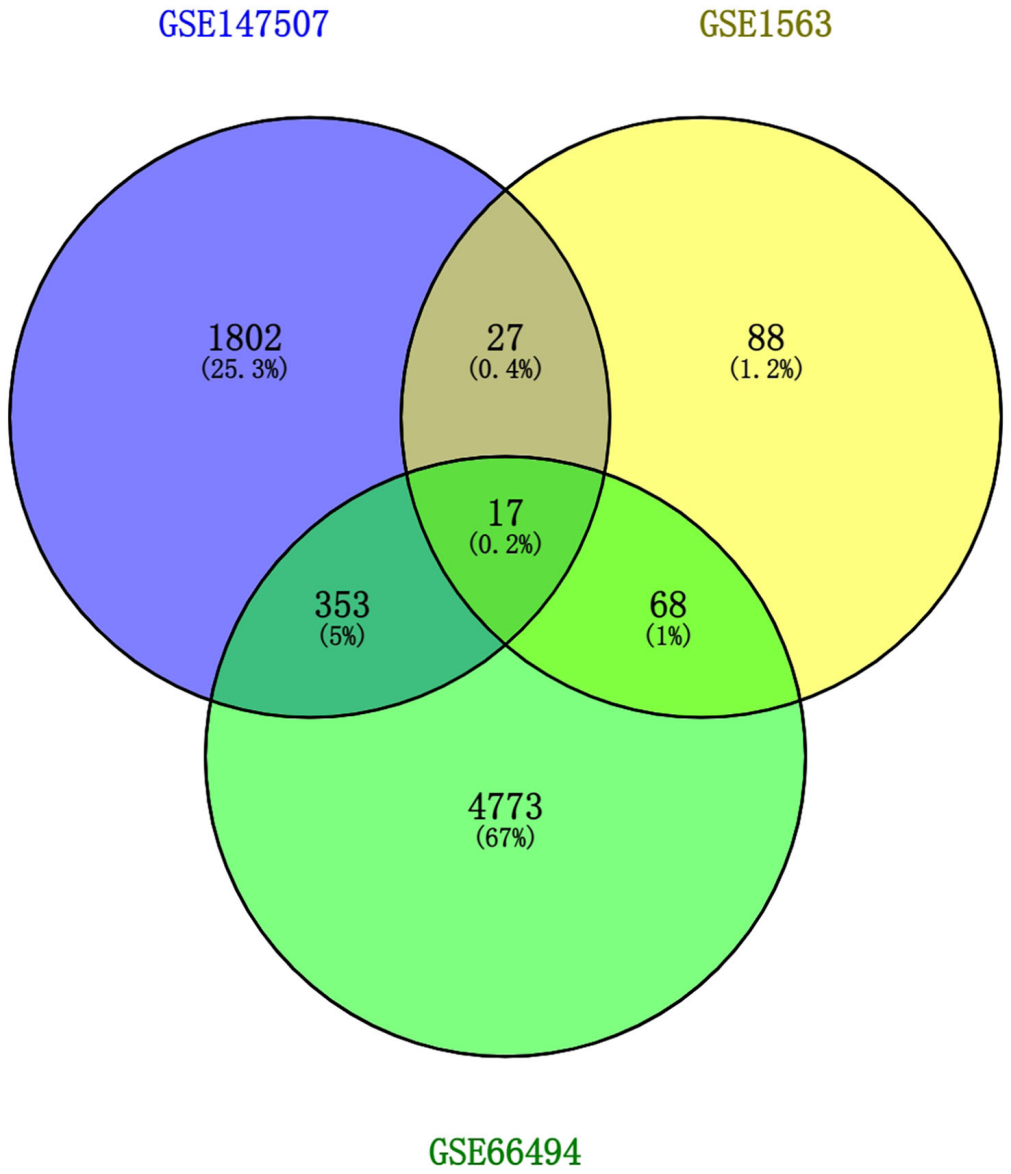
This study incorporates two microarray datasets and one RNA-seq dataset, which together encompass AKI (GSE1563), CKD (GSE66494), and SARS-CoV-2 (GSE147507). This integrated analysis identified 17 DEGs that are common to SARS-CoV-2, AKI, and CKD. AKI, acute kidney injury; CKD, chronic kidney disease; SARS-CoV-2, severe acute respiratory syndrome coronavirus 2; DEGs, differentially expressed genes.

To discover the interrelationships and implications of AKI and CKD with COVID-19, we analyzed human RNA-seq and microarray datasets from NCBI to classify DEGs related to COVID-19, AKI, and CKD. We assessed the RNA-seq and microarray dataset experiments in the R language environment using the DESeq2 and limma packages with the Benjamin–Hochberg false discovery rate. In total, we identified 2199 genes differentially expressed in COVID-19, and we also detected the most significant DEGs for AKI and CKD: 200 in the AKI dataset and 5,211 in the CKD dataset. All significant DEGs were extracted on the basis of p-value<0.05 and |logFC| ≥ 1. After performing cross-comparative analysis with Jvenn, a reliable web portal for Venn analysis, 17 common DEGs from the AKI, CKD, and SARS-CoV-2 datasets were identified, including HBD, HBB, TANK, RNF6, TAB2, WTAP, PNRC1, ING3, TNFAIP8, S1PR1, SEC24A, NRIP1, MARCKS, BHLHE40, DUSP6, EIF2AK2, and RASGRP1. The expression levels of these 17 common DEGs based on the three datasets are shown in heatmaps (**Supplementary Figure 1**). However, the upregulation and downregulation of these 17 DEGs in the cluster heatmaps of the three diseases were not completely consistent. Overall, these genes may be affected by certain pathways, resulting in inconsistent upregulation and downregulation, and we will further investigate how the upregulation and downregulation of these genes are affected in future studies. The three diseases correlate with each other because they share one or more common genes (46) (**Figure 2**).

### 3.2 Gene Ontology and pathway enrichment analyses

**Figure 3:**
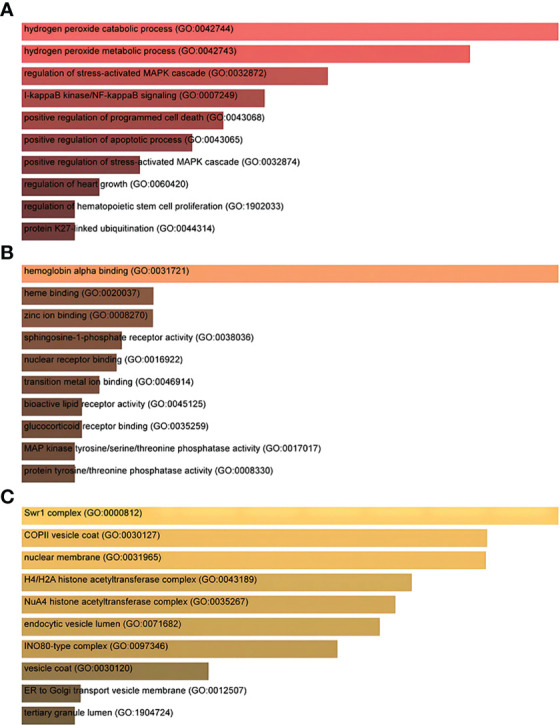
The ontological bar graphs of the DEGs that are shared among SARS-CoV-2, AKI, and CKD using the Enricher online tool. The GO function is divided into three parts: **(A)** biological processes, **(B)** molecular function, and **(C)** cellular component. Each bar graph represents a function in GO. DEGs, differentially expressed genes; SARS-CoV-2, severe acute respiratory syndrome coronavirus 2; AKI, acute kidney injury; CKD, chronic kidney disease; GO, Gene Ontology.

**Figure 4:**
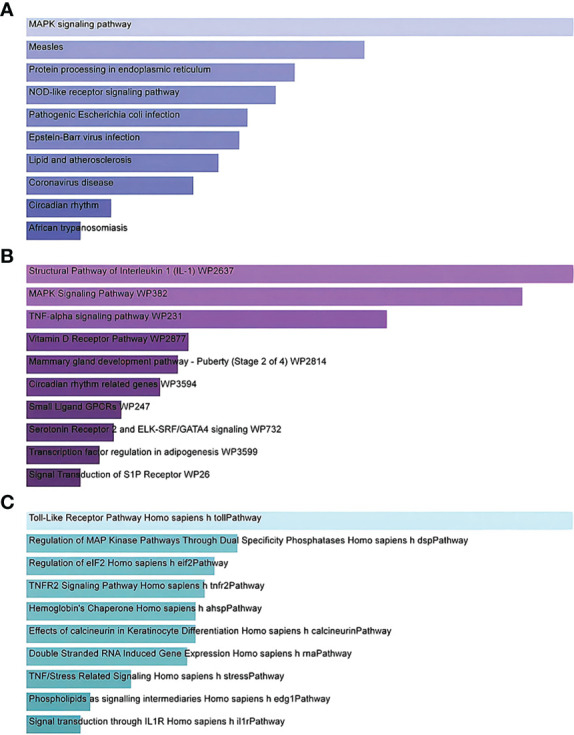
Bar graphs showing pathway enrichment analysis of DEGs shared by SARS-CoV-2, AKI, and CKD as performed by Enricher: **(A)** KEGG 2019 human pathway, **(B)** WikiPathways, and **(C)** BioCarta. Each bar represents a pathway in KEGG/WikiPathways/BioCarta. DEGs, differentially expressed genes; SARS-CoV-2, severe acute respiratory syndrome coronavirus 2; AKI, acute kidney injury; CKD, chronic kidney disease; KEGG, Kyoto Encyclopedia of Genes and Genomes.

Gene Ontology and pathway enrichment analyses were used to identify the biological significance and enriched pathways for the shared DEGs. Gene Ontology analysis is performed within three categories (biological process, cellular component, and molecular function) (**Figures 3A–C**); pathway analysis reveals the functional pathways in which genes are enriched. The most affected pathways of the DEGs common to AKI, CKD, and COVID-19 were gathered from three global databases, including KEGG, WikiPathways, and BioCarta. The top 10 pathways in WikiPathways include the structural pathway of interleukin 1 (IL-1), MAPK signaling pathway, TNF-α signaling pathway, vitamin D receptor pathway, mammary gland development pathway–puberty (Stage 2 of 4), circadian rhythm-related genes, small ligand GPCRs, serotonin receptor 2 and ELK-SRF/GATA4 signaling, transcription factor regulation in adipogenesis, and signal transduction of the S1P receptor. The top 10 pathways in KEGG were the MAPK signaling pathway, measles, protein processing in the endoplasmic reticulum, NOD-like receptor signaling pathway, pathogenic *Escherichia coli* infection, Epstein–Barr virus infection, lipid and atherosclerosis, coronavirus disease, circadian rhythm, and African trypanosomiasis. The top 10 pathways in BioCarta include the Toll-like receptor pathway, regulation of MAP kinase pathways through dual specificity phosphatases, regulation of elF2, TNFR2 signaling pathway, hemoglobin’s chaperone, effects of calcineurin in keratinocyte differentiation, double-stranded RNA-induced gene expression, TNF/stress-related signaling, phospholipids as signaling intermediaries, and signal transduction through lL1R (**Figures 4A–C**).

### 3.3 Classification of hub proteins and submodules

**Figure 5:**
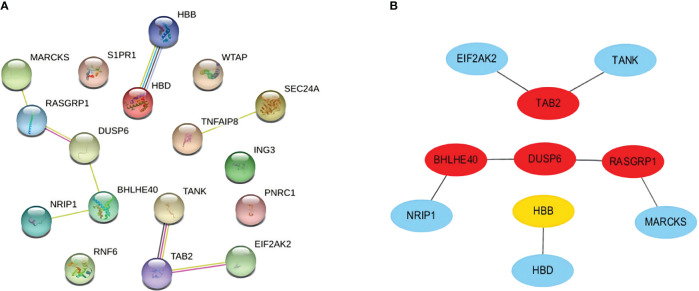
**(A)** PPI network with nodes representing DEGs and edges representing interactions between nodes among SARS-CoV-2, AKI, and CKD. **(B)** Determination of hub genes from the PPI network by using the cytoHubba plugin in Cytoscape. The latest MCC procedure of the cytoHubba plugin was pursued to obtain hub genes. Here, the red nodes indicate the highlighted top 4 hub genes and their interactions with other molecules. PPI, protein–protein interaction; DEGs, differentially expressed genes; SARS-CoV-2, severe acute respiratory syndrome coronavirus 2; AKI, acute kidney injury; CKD, chronic kidney disease; MCC, maximal clique centrality.

We carefully checked the PPI network from STRING and visualized it in Cytoscape to predict common DEG interactions and related pathways. The majority of interconnected nodes are considered hub genes of a PPI network. Based on PPI network analysis incorporating the cytoHubba plugin in Cytoscape, we classified the top 4 DEGs as the most influential genes; DUSP6, BHLHE40, RASGRP1, and TAB2 were detected as hub genes (**Figure 5A**).

These hub genes are potential biomarkers, and the results may lead to new therapeutic strategies to study diseases. As hub genes are potential markers, we also constructed a submodule network with the cytoHubba plugin to better understand their near connectivity and proximity (**Figure 5B**).

### 3.4 Determination of regulatory signatures

**Figure 6:**
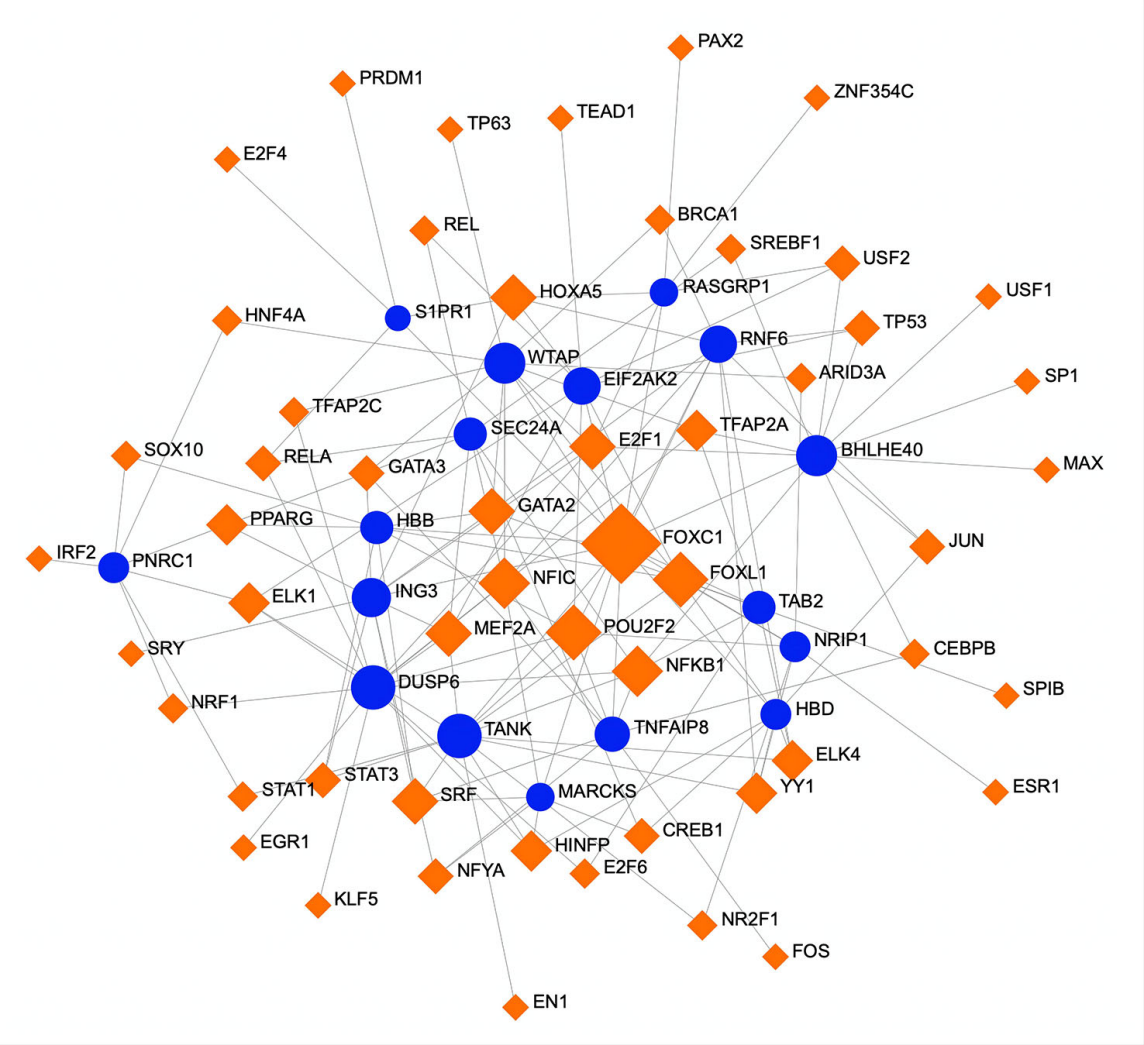
A regulatory interaction network of DEG–TFs derived from NetworkAnalyst. Here, the square nodes represent TFs, and gene symbols are circled as they interact with TFs. The larger the square or circle, the more important the TFs or DEGs are in this network. DEG, differentially expressed gene; TFs, transcription factors.

**Figure 7:**
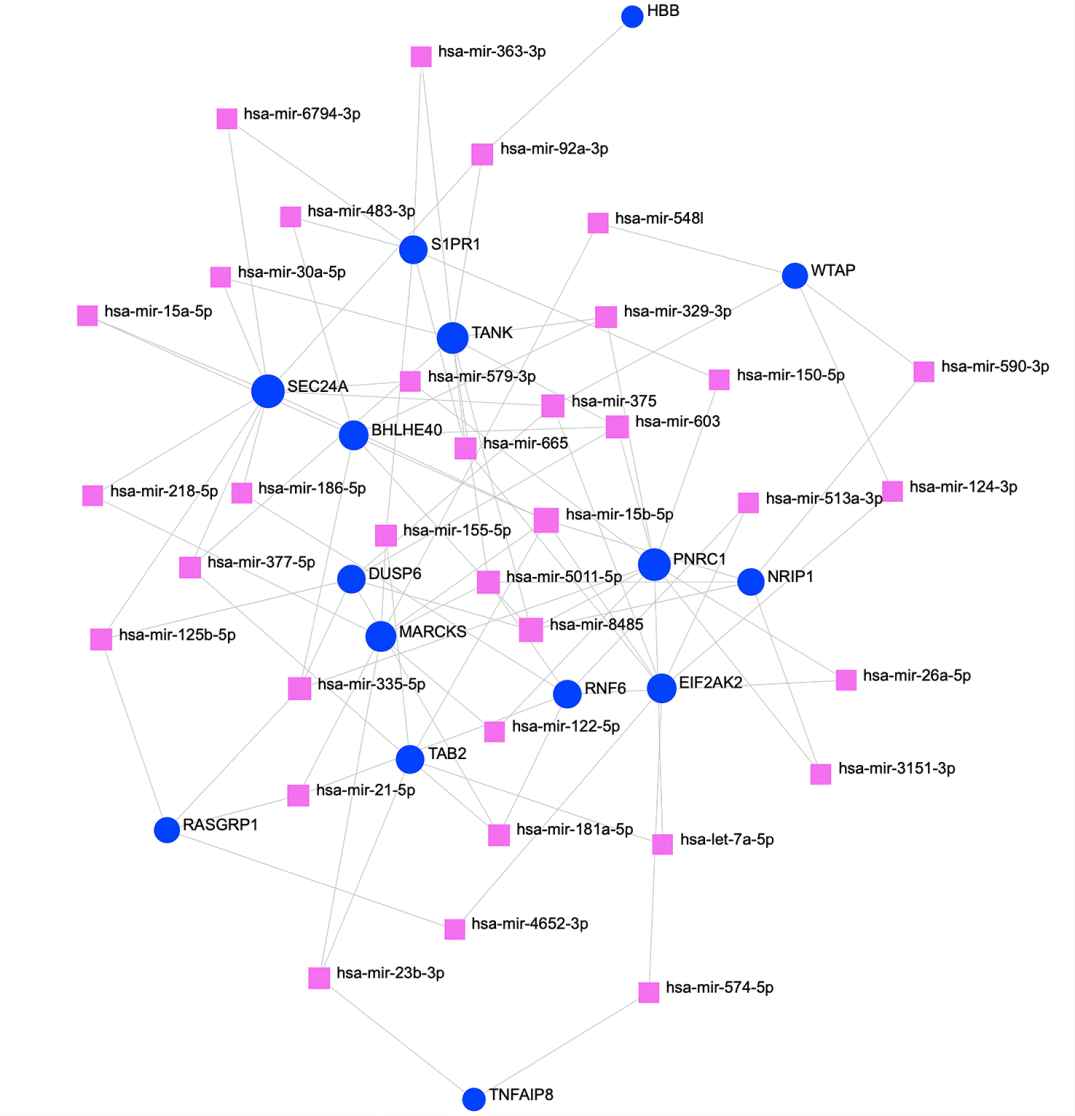
The regulatory interaction network of DEGs and miRNAs. The square nodes represent miRNAs, and gene symbols interact with miRNAs as circles. The larger the square or circle, the more important the miRNAs or DEGs are in this network. DEGs, differentially expressed genes.

To determine substantial changes at the transcriptional level and understand the hub proteins’ regulatory molecules and common DEGs, we adopted a network-based approach to decode regulatory TFs and miRNAs. From TF–gene and miRNA–gene interaction network analyses, it was ascertained that 53 TF (**Figure 6**) and 34 posttranscriptional miRNA (**Figure 7**) regulatory signatures are regulated by more than one common DEG, indicating that they strongly interact with each other.

### 3.5 Identification of candidate drugs

Evaluating protein–drug interactions is crucial to understand the structural features recommended for receptor sensitivity (46, 47). Regarding common DEGs as potential drug targets in AKI, CKD, and COVID-19, we identified eight possible pharmaceutical molecules based on transcriptome signatures from the DSigDB database using Enrichr. A list of the top 8 chemical compounds for three diseases according to p-value and potential drugs for DEGs, including common chemical compounds, is presented in **Table 1**.

**Table 1:** List of the suggested drugs for COVID-19 with AKI or CKD.

	Name	Adjusted p-value	Odds ratio	Combined score
1	**Tanespimycin** ssMCF7 DOWN	0.000926	203.69	2809.91
2	**Camptothecin** MCF7 DOWN	0.001249	10.91	122.15
3	**Niclosamide** HL60 UP	0.001249	35.03	393.6
4	**Camptothecin** PC3 DOWN	0.001249	11.06	124.87
5	**Pyrvinium** MCF7 UP	0.001249	36.73	419.4
6	**Staurosporine** MCF7 DOWN	0.001249	16.41	188.24
7	**Dimethyloxalylglycine** PC3 UP	0.001249	94.94	1107.22
8	**Sulpiride** PC3 DOWN	0.001249	17.29	203.43
9	**Daunorubicin** MCF7 DOWN	0.001249	12.97	160.49
10	**Niclosamide** MCF7 UP	0.001249	51.36	652.36

MCF and PC3 represent different cell lines. Adjusted p-value<0.05 has a statistical difference.

AKI, acute kidney injury; CKD, chronic kidney disease.

**Figure 8:**
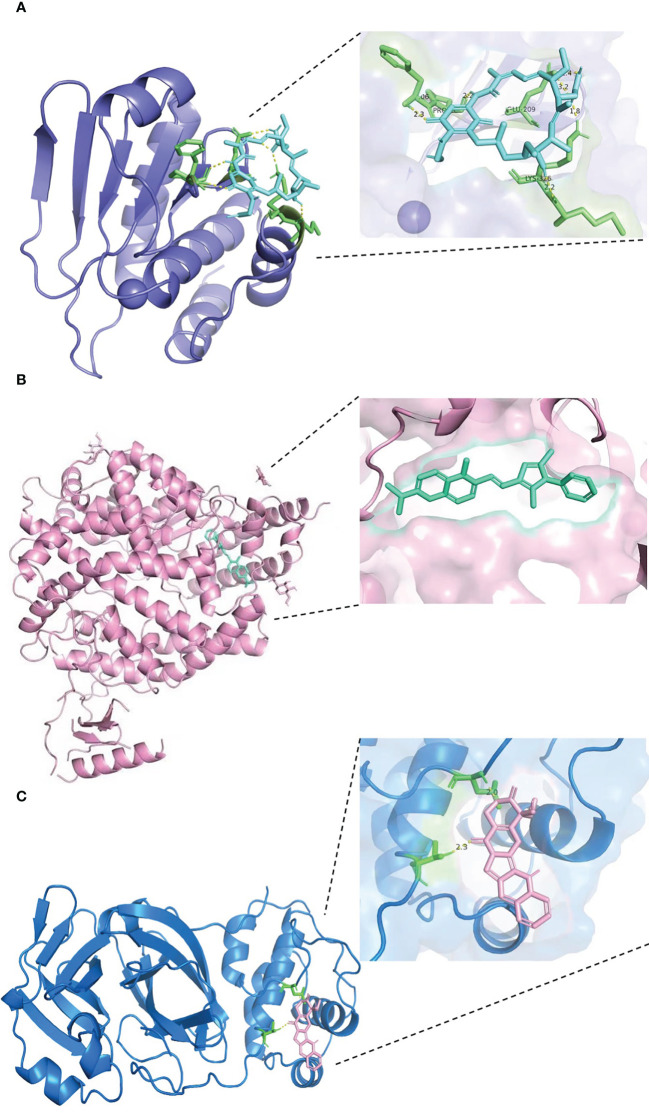
Results of molecular docking of drug and protein. Each figure shows the overall picture of the docking of protein and drug molecules and the enlarged picture of the docking part. **(A)** The docking diagram of DUSP6 with tanespimycin, with binding energy of −5.67 kcal/mol. **(B)** The binding energy between ACE2 and pyrvinium is −6.61 kcal/mol. **(C)** 3CLpro and camptothecin docking diagram, with binding energy of −5.59 kcal/mol.

The results of docking analyses are shown in **Supplementary Table 1**, in which lower binding energy indicates a more stable docking result. The docking results for pyrvinium with BHLHE40, RASGRP1, and ACE2 are the most stable, with binding energies of −7.46, 7.77, and −6.61 kcal/mol, respectively. The most stable drug molecules docked with DUSP6 and TAB2 are tanespimycin and niclosamide, with binding energies of −5.67 and −9.26 kcal/mol, respectively; the most stable drug binding to 3CLpro is camptothecin, with a binding energy of −5.59 kcal/mol (**Figure 8**).

### 3.6 MM/GBSA results

Based on the trajectory of the molecular dynamics simulation, we used the MM/GBSA method to calculate binding energy, which can accurately reflect the binding effect of a drug molecule and target protein.

As shown in **Supplementary Table 2**, the binding energies of drug molecule ligands and proteins in the tanespimycin–DUSP6, pyrvinium–RASGRF1, niclosamide–TAB, pyrvinium–BHLHE40, pyrvinium–ACE2, and camptothecin–3CLpro systems were found to be −15.7857 ± 1.3991, −27.6909 ± 0.9977, −14.8572 ± 0.5838, −21.5866 ± 0.9644, −28.5042 ± 1.4538, and −13.2160 ± 1.4146, respectively. Negative values indicate that the two molecules have binding affinity for the target protein, and lower values indicate stronger binding. Obviously, our calculations show that all systems have the potential to bind, with the binding affinities of pyrvinium–BHLHE40 and pyrvinium–TAB2 being significantly lower than 20 kcal/mol, suggesting that the two complexes have better binding effects. Through energy decomposition, we can determine that in the pyrvinium–ACE2 complex, the electrostatic energy (EEL) has a strong contribution; in contrast, the electrostatic energy has a weak contribution in the tanespimycin–DUSP6 and camptothecin–3CLpro complexes. The van der Waals energy (VDW) plays a role in all combinations. In addition, the polar solvation energy of 24 is 331.4498 ± 1.3050 kcal/mol, indicating that it is not conducive to binding, with the non-polar solvation energy playing a weak role. The remaining polar or non-polar solvation energy contribution of the other systems is not significant and has little effect on binding.

### 3.7 Identification of disease association

**Figure 9:**
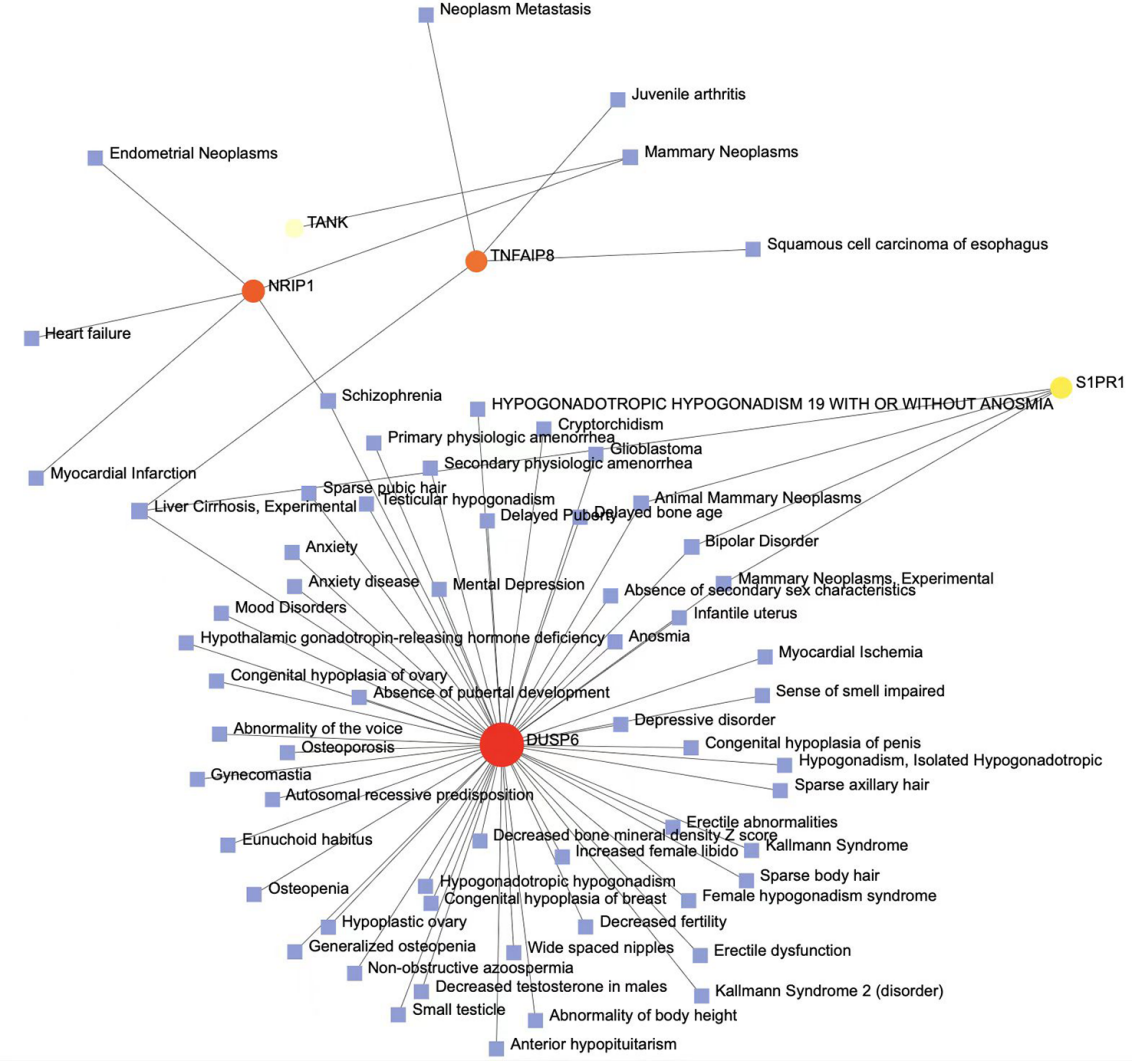
The gene–disease association networks show diseases associated with mutual DEGs. Diseases are represented by square nodes, and their associated gene symbols are represented by circular nodes. The larger the square or circle, the more important the diseases or DEGs are in this network. DEGs, differentially expressed genes.

Different diseases can correlate with each other and usually share one or more similar genes (46). Therapeutic design strategies for combating disease have begun to uncover relationships between genes and disorders (48). According to NetworkAnalyst, studies have reported an impaired sense of smell, heart failure, testicular hypogonadism, and mood disorders associated with COVID-19. Persistent loss of smell or taste without an obvious cause (e.g., typhoid) is called olfactory failure. The most common causes of olfactory loss are allergic sinusitis, nasal polyps, colds, and viral infection. Heart failure is a syndrome of impaired cardiac circulation due to impaired systolic or diastolic function, which is not an independent disease but rather the end stage of various heart diseases, resulting in blood stagnation in the venous system and inadequate perfusion in the arterial system. In the majority of heart failure cases, the common initial manifestation is pulmonary congestion. In addition, many COVID-19 patients experience symptoms of renal tissue ischemia, which probably progresses to AKI or even CKD (**Figure 9**).

### 3.8 COVID-19 diagnostic model

The receiver operating characteristic (ROC) values of each machine learning model for training and validation sets are shown in **Supplementary Figures 2A, B**, respectively. Calibration curves for each model are shown in **Supplementary Figure 2C**. XGBoost had the highest AUC in both the training set and validation set at 1 and 0.792, respectively (**Supplementary Figures 2A, B**). The calibration plot in **Supplementary Figure 2C** shows that the XGBOOST model was also the most accurate. **Supplementary Tables 3**, **4** show that the AUC, cutoff, accuracy, sensitivity, specificity, positive predictive value, negative predictive value, and F1 score of XGBoost for the training set were 1.000, 0.683, 0.984, 1.000, 1.000, 1.000, 0.978, 1.000, and 0.961, respectively. In brief, XGBoost was the best-performing model, and we used it to build a diagnostic model for COVID-19.

After filtering out the best-performing XGBoost model, we used the “SHAP” package to explain the importance of key variables to the model. **Supplementary Figure 2D** shows the contribution of each variable, with red dots indicating positive contributions and blue dots indicating negative contributions. A shorter distance from the point to the left indicates a smaller value and a larger value at a longer distance. For example, a higher expression value of TANK predicts a higher risk of COVID-19, whereas a lower value predicts a lower risk.

## 4 Discussion

AKI and CKD are currently considered to be two stages of renal disease progression; the former is a common complication and mortality risk factor in COVID-19 patients, and the latter is an independent risk factor for COVID-19 and poor prognosis of COVID-19 (49, 50). Decreased GFR is strongly associated with the prevalence and mortality of COVID-19, and chronic metabolic diseases resulting in CKD, such as diabetes, hypertension, and obesity, are also related to COVID-19 mortality (9). In this study, we collected three datasets and used a computational network data analysis method to discover gene expression patterns and molecular pathways of AKI, CKD, and COVID-19 and identify molecular targets of potential biomarkers, providing more treatment options for different disease conditions (51–53). By analyzing transcriptional profiles of SARS-CoV-2, AKI, and CKD to identify genes with altered expression in SARS-CoV-2 infection implicated in the pathogenesis of AKI and CKD, we report novel interaction mechanisms. Seventeen common DEGs were revealed that showed similar expression patterns in the three diseases and were evaluated by Gene Ontology (GO) pathway analysis functions based on p-values to acquire insight into the pathophysiology of AKI, CKD, and COVID-19.

GO involves a genetic adjustment context based on a general theoretical model that promotes genes and their internal relationships. Evolutionary studies have gradually provided biological knowledge of genetic functions and their regulation in different ontological categories (54). From Enrichr, three categories of GO analysis, namely, biological process (molecular activities), molecular function (activities at the molecular level), and cellular component (genes that regulate function), were evaluated through the GO database as a source of annotation for ontological processes (55). In the biological process category, hydrogen peroxide catabolic and hydrogen peroxide metabolic processes were among the top GO terms. Hydrogen peroxide has emerged as a major redox metabolite that functions in redox sensing, signaling, and redox regulation (56). Hydrogen peroxide catabolism contributes to limiting or repairing oxidative damage (57). SARS-CoV-2-infected individuals are susceptible to oxidative stress, and their ability to resist oxidative stress may be associated with the inflammatory status and may have little association with the severity of the disease (58). One study revealed that SARS-CoV-2 captures iron and generates reactive oxygen species to injure the human immune system while promoting the catabolism of hydrogen peroxide to oxygen and water in phagocytes to reduce killing capacity (59). Excessive peroxide causes a renal oxidative stress response, inducing mitochondrial metabolism and kinetic dysfunction and causing inflammation and apoptotic cell death, which induce AKI and aggravate CKD (60). Chen et al. and Huang et al. found massive infiltration of CD4+ T cells, CD56+ natural killer cells, and CD68+ macrophages in the tubular stroma in the renal tissue of COVID-19 patients and that activated T cells migrate to the location of infection to exert their function (61, 62). Under these conditions, SARS-CoV-2 may promote necrosis or apoptosis of T cells by activating reactive oxygen species metabolism, consequently hindering viral clearance, and excess peroxide production can trigger the oxidative stress response in kidney tissue, causing inflammation, cell death, and the deterioration of renal function. Regarding molecular function, hemoglobin-α binding and heme-binding activity were the two top GO pathways. Endothelial cell expression of hemoglobin-α regulates nitric oxide signaling, impacting blood perfusion and oxygen supply (63). Kronstein-Wiedemann et al. found that SARS-CoV-2 infects red blood cell progenitors and dysregulates hemoglobin and iron metabolism, impairing hemoglobin homeostasis and exacerbating COVID-19 (64). It has also been demonstrated that an abnormal hemoglobin phenotype is directly associated with a decreased renal function (65). Moreover, free heme is a pro-oxidant that can disrupt homeostasis *in vivo* through proinflammatory and cytotoxic effects (66). AKI causes renal hemopexin accumulation, potentially impacting heme Fe-mediated tubular injury and leading to disease progression (67). Therefore, it cannot be ruled out that SARS-CoV-2 infection may initiate AKI by disrupting hemoglobin metabolic homeostasis, which in turn can aggravate this vicious cycle, leading to sustained progression of renal function impairment.

Pathway analysis is a key step to reflect the internal reaction process of an organism with a viral infection. KEGG, WikiPathways, and BioCarta pathways of 17 common DEGs were identified to find similar pathways for AKI, CKD, and COVID-19. Our analysis found that the MAPK signaling pathway, the structural pathway of IL-1, and the Toll-like receptor pathway may have pivotal roles in the occurrence mechanisms of these three diseases. The MAPK signaling pathway activated in viral infections links cell-surface receptors to the transcription machinery, transducing extracellular signals into several outputs, which may also affect the mechanisms of host defense and apoptosis (68). A variety of studies have demonstrated that the MAPK signaling pathway is associated with cell injury, inflammation, and fibrosis, all of which result in acute and chronic kidney diseases (69–73). Weckbach et al. and Saheb et al. found that MAPK pathway activation is one of the important mechanisms of organ inflammation in SARS-CoV-2 infection and may affect sensitivity to steroid treatment (74, 75). In general, the MAPK pathway is vital for regulating organ inflammation and function and is probably involved in the occurrence of multiple-organ dysfunction in COVID-19 patients. COVID-19 is suggested to involve a proinflammatory factor pattern similar to that of some autoimmune diseases; therefore, a potential way to treat COVID-19 may be by inhibiting increases in cytokine and chemokine levels (76). IL-1 binds to specific receptors, which leads to increases in coreceptor and intracellular signal conduction, thereby inducing an effective inflammatory response (77). In the chronic inflammatory mechanism underlying the progression of AKI to renal fibrosis, IL-1 signaling plays an important role (78, 79). Following secretion of chemokines and cytokines such as IL-1β, IL-6, TNF-α, IL-21, and IL-8, the SARS-CoV-2-induced cytokine storm and hyperinflammatory response have pivotal roles in infection severity, AKI development, and death (80). Bowe et al. even pointed out that survival in COVID-19 somehow predisposes patients to worsening subsequent long-term kidney function (81). Toll-like receptors (TLRs) are activated by foreign and host molecules to initiate the immune response. TLR agonists are able to serve as a possible therapeutic agent or a vaccine adjuvant for cancers or infectious diseases; TLR inhibitors may be a promising approach to the treatment of autoimmune diseases and bacterial and viral infections (82). In AKI caused by ischemia and reperfusion, researchers have discovered that proximal tubule TLR4 expression is linked to inflammation and apoptosis following hypoxia–reoxygenation injury (83). Activation of TLR4 signaling regulates the transcription of numerous proinflammatory cytokines and chemokines, resulting in renal inflammation (84, 85). Therefore, the Toll-like receptor pathway is involved in the pathogenesis of SARS-CoV-2 infection and kidney diseases. Nevertheless, the mechanism by which SARS-CoV-2 triggers inflammation is not clear. Recently, a study discovered that antibody-mediated SARS-CoV-2 uptake by monocytes and macrophages causes inflammatory cell death that eliminates the production of infectious viruses and results in systemic inflammation that contributes to COVID-19 pathogenesis. This strong inflammatory effect may be the main cause of severe illness and death (86). The underlying inflammatory pathways identified in these three diseases once again demonstrate that inflammation is a significant mechanism by which SARS-CoV-2 infection leads to damage in multiple organs.

Based on the analysis of DEGs, we established a PPI network showing protein biology and predicting relevant drug targets at the proteomic level and identified hub proteins expressed by topology metrics that may serve as biomarkers or key treatment targets of COVID-19 and are associated with various pathobiological mechanisms. The top hub proteins represent different diseases, most of which are risk factors for AKI, CKD, and COVID-19. The top 4 topological metric hub proteins (DUSP6, BHLHE40, RASGRP1, and TAB2) are clearly involved in these diseases. In this step, the cutoff (parameter) of the topological metric for hub proteins is 15 (degree). DUSP6, a negative regulator of the extracellular signaling-regulated kinase (ERK) signaling pathway, is a broadly expressed dual-specificity phosphatase protein and has roles in apoptosis inhibition and cellular protection (87, 88). Han et al. found that H_2_O_2_ potentially promotes heart regeneration in zebrafish by stimulating MAPK signaling through a depression mechanism involving DUSP6 (89). Missinato et al. also suggested that DUSP6 attenuates Ras/MAPK signaling during regeneration and that suppressing DUSP6 can enhance cardiac repair (90). In contrast, dual inactivation of DUSP4 and DUSP6 selectively impairs growth in NRAS and BRAF mutant cells in cancer through hyperactivation of MAPK signaling (91). These studies demonstrate that DUSP6 plays a vital role in tissue damage and repair by regulating hydrogen peroxide metabolism and the MAPK signaling pathway. Moreover, in diabetic nephropathy patients who have the highest prevalence of CKD, DUSP6 has been found to mediate protection against high glucose-induced inflammation (92). Interestingly, Hsu et al. demonstrated that DUSP6 also plays a positive role in the pathological process of endothelial inflammation through TNF-α-induced endothelial intercellular adhesion molecule-1 (ICAM-1) expression, a process that is independent of ERK signaling (93). Expression of ACE2 in vascular endothelial cells provides the pathophysiological basis for viral invasion. Histopathological examination of COVID-19 patients has revealed that SARS-CoV-2 directly invades endothelial cells, causing diffuse endothelial cell inflammation and microvascular damage, which most likely leads to the failure of multiple organs, including the kidneys (94, 95). Hence, manipulation of DUSP6 holds great potential for the treatment of acute inflammatory diseases, such as AKI and COVID-19. There are more studies on DUSP6 in oncology, demonstrating that its expression improves tumor proliferation and drug resistance (96–99). The factor BHLHE40 has emerged as an important regulator of immunity during infection, autoimmunity, and inflammatory conditions, especially in cytokine production and proliferation (100). BHLHE40 also plays an important role in the transcriptional regulation of immune cell infiltration (101). As mentioned above, the cytokine storm and infiltration of immune cells in tissues and organs are pivotal causes of the aggravation and organ dysfunction occurring in COVID-19. As an important immune regulator, BHLHE40 is significant in regional and systemic inflammatory responses to AKI, CKD, and SARS-CoV-2 infection. Feng et al. identified that 17β-estradiol (E2) regulates BHLHE40 expression to exert a protective effect on carotid artery ligation and that upregulation of BHLHE40 in vascular smooth muscle cells (VSMCs) results in suppression of MAPK signaling (102). One study found that BHLHE40 plays an important role as a transcription factor in autoreactive T helper (Th) cell pathogenicity. Lin et al. showed that BHLHE40 expression induced by the IL-1 signaling pathway can identify encephalitogenic Th cells and defines a pertussis toxin (PTX)-IL-1-BHLHE40 pathway active in autoimmune neuroinflammation (103). In addition, Camponeschi et al. indicated that B-cell receptor (BCR) or TLR9 activation induces expression of BHLHE40, a key negative regulator of activation-induced proliferation of human B cells and highly expressed in anergic cells (104). Therefore, as a regulator of many significant immune-inflammatory signaling pathways, BHLHE40 participates in the pathogenic process of immune-inflammatory diseases. RASGRP1 is an important guanine nucleotide exchange factor and activator of the RAS-MAPK pathway following T-cell antigen receptor (TCR) signaling, and its deficiency causes immunodeficiency with impaired cytoskeletal dynamics (105). Moreover, Zhang et al. found that RASGRP1 mediates TLR2-induced ERK1/2 activation and inhibition of IL-12p40 production, which regulates TLR9 activation to induce an appropriate protective IL-12 response (106). By promoting lymphocyte proliferation, RASGRP1 activity is also indispensable to autoimmunity (107). Thus, RASGRP1 activity is essential to the innate protective immune response. In addition, a study discovered that its expression in vascular endothelial cells maintains vascular health (108). Based on this evidence, RASGRP1 has great potential to become a pivotal regulatory target of COVID-19 with AKI and CKD. Nuclear TAB2 is a repressor of NF-κB-mediated gene regulation. The TAB2 protein is expressed in the vascular endothelium of most tissues (109), and its downregulation has a significant effect in inhibiting the inflammatory response and protecting tissue from acute injury, and it can serve as a target of manipulation for multiple cytokines (110, 111). TAB2 gene may be one of the target genes for COVID-19 infection and organ injury. Taken together, we reveal that the top 4 hub genes are all involved in the regulation of microvascular endothelial cell function. Many published studies support that endothelial inflammation is the key mechanism promoting COVID-19 progression and multiorgan dysfunction. Therefore, the hub genes identified in this study are potential biomarkers and therapeutic targets for COVID-19.

Transcriptional and posttranscriptional modifications are important aspects of epigenetics, influencing gene expression. Therefore, we analyzed TF–gene and miRNA–gene interactions to identify the transcriptional and posttranscriptional regulators of common DEGs. TFs control transcriptional processes and proportions, and miRNAs play key roles in gene regulation at the posttranscriptional level and in RNA silencing. The discovery of relationships between DEGs, TFs, and miRNAs is conducive to an understanding of the molecular-level progression of diseases. The identified TFs, such as FOXC1, FOXL1, POU2F2, NFIC, NFkB1, MEF2A, GATA2, and E2F1, are mainly associated with different types of cancers and congenital disorders. DUSP6, BHLHE40, RASGRP1, and TAB2, the top 4 topological metric hub genes, appear to be pivotal molecular targets of COVID-19, AKI, and CKD. The TF–gene interaction network indicates that FOXC1 is involved in BHLHE40, RASGRP1, and TAB2 expression but that FOXL1 only regulates TAB2. Additionally, POU2F2, NFIC, NFkB1, and MEF2A regulate the expression of DUSP6 gene, and NFkB1 manipulates the transcription of BHLHE40 and TAB2 genes. GATA2 is involved in TAB2 expression, and E2F1 regulates the expression of the other three hub genes. In detail, FOXC1 and FOXL1 belong to the human Forkhead-box (FOX) gene family, which is widely involved in cellular activities (112). For example, Koo et al. reported that FOXC1 appears to contribute to pathological angiogenesis by regulating vascular endothelial growth factor signaling (113). Additionally, a study by Zhang et al. showed that FOXC1, as an ischemia-inducible TF, upregulates the expression of TLR members in myocardial ischemia, promoting cardiac inflammation and playing a detrimental role in myocardial ischemia (114). Although studies of FOX genes in infection and kidney disease are scarce, current evidence suggests that FOXC1 activates inflammation under hypoxia, which may have a regulatory role in SARS-CoV-2 infection and renal dysfunction. POU2F2 is a member of the POU transcription factor family and is involved in the immune response by regulating B-cell proliferation and differentiation genes (115). NFIC belongs to the family of transcription factors involved in various morphogenetic processes during development (116). A number of studies have found that NFIC controls cell proliferation by regulating TGF-β1 signaling in adult regenerative processes, such as tooth root development, hair follicle cycling, and hepatocyte proliferation (117–119). Overall, the roles of these transcription factors in kidney disease and infection have been poorly investigated to date. MiRNAs are small non-coding RNAs that serve as central players that regulate the posttranscriptional processes of gene expression. They bind to target mRNAs and repress their translation by inducing their degradation or inhibiting their translation to control mRNA expression. RNA sequencing is becoming popular in the postgenomic era, but high-throughput experimental technologies for miRNA target identification are still expensive and time-consuming. Therefore, an increasing number of bioinformatics approaches are being developed for miRNA studies, especially for miRNA target prediction. In this study, we successfully used bioinformatics tools to accurately identify miRNAs targeting the DEGs of the three diseases. Some of these miRNAs are closely related to regulating the expression of the hub genes. For instance, hsa-mir-181a-5p, hsa-mir-125b-5p, and hsa-mir-603 participate in the expression of the DUSP6 gene; both hsa-mir-329-3p and hsa-mir-335-5p are associated with BHLHE40 expression, and hsa-mir-125b-5p, hsa-mir-335-5p, and hsa-mir-21-5p are involved in RASGRP1 expression; hsa-mir-181a-5p, hsa-mir-155-5p, and hsa-mir-21-5p manipulate TAB2 expression. Specifically, miRNA mutations often lead to the development of various diseases. Some miRNAs are involved in lung cancer (e.g., hsa-mir-665, hsa-mir-30a-5p, hsa-mir-150-5p, and hsa-mir-181a-5p) (120–123), immune disorders (e.g., hsa-mir-92a-3p, hsa-mir-665, and hsa-mir-155-5p) (124–126), and different types of chronic inflammation or infection (e.g., hsa-mir-483-3p, hsa-mir-92a-3p, and hsa-mir-335-5p) (127–129). Most miRNAs are related to cancer and congenital diseases, though some specific miRNAs are related to the pathogenesis of AKI. For example, Zhang et al. discovered that miR-181a-5p inhibits pyroptosis through the downregulation of NEK7 in lipopolysaccharide (LPS)-induced HK-2 cells and cecum ligation and puncture (CLP)-induced mice and indicated that miR-181a-5p is a new potential therapeutic target for sepsis-induced AKI therapy (130). The research results of He et al. confirmed that miR-122 directly targets vitamin D receptor (VDR) in renal tubular cells, which strongly suggests that miR-122 upregulation contributes to LPS-induced kidney injury by downregulating VDR expression (131). Moreover, hsa-mir-122-5p has been proven to regulate the ASF1A, BRWDM, and PFKFB2 signaling pathways, a potential mechanism for the development of AKI in transplanted kidneys (132). As a novel small molecule, miR-665-3p regulates autophagy by targeting ATG4B, indicating that miR-665-3p inhibition is a potential therapeutic approach against inflammation and apoptosis for the treatment of ischemia–reperfusion (133). hsa-miR-483-3p is associated with diabetic renal vascular injury and lupus nephritis (134), and hsa-mir-186-5p is involved in a variety of acute organ injury processes (135). Accordingly, TFs and miRNAs target major proteins to alter particular diseases (136). SARS-CoV-2 infection possibly induces transcriptional regulator mutations regulating primary signaling pathways, thus activating inflammatory responses and leading to impairment of renal function.

Mutations in genes are often closely related to multiple diseases, and we performed gene–disease (GD) analysis to predict associations between significant DEGs and various diseases. The results revealed various diseases from the common DEGs of AKI, CKD, and COVID-19, which include DUSP6, NRIP1, TNFAIP8, S1PR1, and TANK. It is notable that most of these diseases are involved in reproductive phylogenetic problems, psychophysiological disorders and cancers, and occasionally heart dysfunctions. DUSP6, as an important DEG, has been discovered to be associated with gonad development, altered sexual signs, and psychophysiological disorders, such as hypogonadism, absence of secondary sex characteristics, and mood and depressive disorders. COVID-19 has a strong relationship with hypogonadism. Recent studies have demonstrated the mechanisms by which secondary immune responses govern endocrine function in SARS-CoV-2 infection and can hinder testosterone synthesis in male patients, affecting male reproductive health; there is also a possibility of inflammation due to the infection, direct viral invasion of the testis, and drug-related damage (137, 138). These findings indicate that men should be considered at higher risk of poor prognosis or death. Anxiety and depression are common manifestations in COVID-19 patients, and the immune system perturbation caused by infection and the roles of inflammatory and clinical predictors may induce psychopathology. The COVID-19 pandemic might be associated with psychiatric disorders (139, 140). TNFAIP8 and NRIP1 are mostly associated with tumors, with breast cancer appearing more frequently in our GD network. Some studies have suggested that estrogen levels in COVID-19 patients can affect the inflammatory state and microbiome, which may be a mechanism of breast tumor production; psychological factors also have certain effects on female patients (141, 142). Additionally, heart diseases, such as heart failure and myocardial infarction, may be regulated by NRIP1. Cardiac injury in COVID-19 patients seems to be associated with higher mortality. Myocardial infarction, cardiomyopathies, arrhythmias, fulminant myocarditis, and venous thromboembolism are the most common cardiovascular complications of COVID-19 (143). Excessive secretion of inflammatory cytokines (IL-6 and TNF-α) leads to systemic inflammation and multiple organ dysfunction syndrome, severely affecting the cardiovascular system (144). Furthermore, SARS-CoV-2 tropism and interaction with the RAAS system may enhance inflammatory responses and cardiac aggression (145). It is clear that COVID-19 is a systemic disease complicated by multiorgan dysfunction, and organ crosstalk plays a key role in this process. The involvement of the kidney, as the main organ leading to organ crosstalk, was first defined in patients with acute respiratory distress syndrome (ARDS) and COVID-19. The lungs and the kidneys cooperate to maintain the electrolyte balance and the acid–base balance in the body, and impairment of renal function disrupts the balance and affects lung function (95). In addition to the lungs, the kidneys engage in crosstalk with multiple other organs. Progression of CKD is also often accompanied by reproductive health challenges, including menstrual abnormalities, impaired sexual health, and reduced fertility (146). Crosstalk between the gonad and the kidney may also be related to reproductive system problems in COVID-19 patients. Depression has been reported to be the most common psychological problem in patients with CKD and is influenced by biological, psychological, and socioeconomic factors (147, 148). Furthermore, psychological symptoms in CKD are independent predictors of adverse clinical outcomes, including faster GFR decrease, dialysis therapy initiation, death, or hospitalization (149). It is possible that the bidirectional relationship between the progression of COVID-19 and depression is also affected by the complex interplay between biopsychosocial factors. Kidney diseases and cancers are intertwined in many ways. On the one hand, underlying kidney disease appears to increase cancer risk and its associated morbidity and mortality (150). Jørgensen et al. showed that an elevated urinary albumin/creatinine ratio at baseline correlates with subsequent cancer incidence (151). Albuminuria is also associated with an increased risk of cancer death from all causes and lung and prostate cancers in men aged 50 and older in the USA (152). As with albuminuria, end-stage renal disease (ESRD) is associated with an increased risk of renal and urinary tract cancer, and increased rates of endocrine cancer, viral infection-related cancer, skin cancer, and liver cancer have also been reported in ESRD patients (153, 154). On the other hand, carcinoma, paraneoplastic renal manifestations, and nephrotoxicity of chemotherapeutic- and molecular-targeted drugs can lead to the development of AKI and sustained impairment of renal function (155). GD analysis demonstrates the common underlying molecular mechanisms of various comorbidities in COVID-19 and kidney disease and highlights a possible reason why the kidney is able to act as the main organ for organ crosstalk in COVID-19.

Currently, some drugs have been approved for the treatment of COVID-19 with few adverse effects. For example, remdesivir and chloroquine have been demonstrated to prevent SARS-CoV-2 infection and COVID-19 (156). Furthermore, baricitinib, which shows antiviral effects by interfering with viral entry into cells, shows improved therapeutic effects in combination with remdesivir (157). Casirivimab and imdevimab (REGN-COV2), neutralizing antibodies, also have shown promising results for SARS-CoV-2 infection by inhibiting viral receptor-binding domain binding to host cells (158). In addition, drugs such as dexamethasone, tocilizumab, and interferon have been shown to have significant effects against COVID-19 (159–161). The protein–drug interaction and molecular dynamics analyses of this study indicate eight possible chemical compounds targeting common DEGs, with different binding affinities for the four hub proteins: DUSP6, BHLHE40, RASGRP1, and TAB2. Pharmaceutical molecules strongly binding to TAB2 were the most abundant, including tanespimycin, camptothecin, niclosamide, pyrvinium, and daunorubicin; molecules strongly binding to RASGRP1 and BHLHE40 were the second most abundant, with the former including camptothecin, niclosamide, and pyrvinium and the latter including tanespimycin, niclosamide, and pyrvinium. The least abundant was the DUSP6 protein, but all pharmaceutical molecules can bind to this protein. We identified the heat shock protein 90 (HSP90) inhibitor tanespimycin as a host-dependent factor of SARS-CoV-2 and an effective, broad-spectrum antiviral drug against human coronavirus (162). Another drug is camptothecin, a quinoline alkaloid originally isolated from the Chinese happy tree, and has been found to have anticancer and antiviral properties. Regarding SARS-CoV-2, camptothecin potentially blocks the interaction of the spike glycoprotein with the ACE2 receptor on host cells (163). Another identified drug is niclosamide, an anthelminthic drug, which is widely used to treat a variety of diseases due to its pleiotropic anti-inflammatory and antiviral activities. An effect *via* interruption of the viral life cycle or induction of the cytopathic effect renders it a possible candidate for COVID-19 (164). Pyrvinium has anthelmintic properties and therapeutic functions against fungi and is a potential novel agent for tumor therapy (165). Daunorubicin belongs to the anthracycline group and is widely used in human cancer chemotherapy (166, 167). Moreover, staurosporine, dimethyloxalylglycine, and sulpiride were found to be potential drugs in this study. Staurosporine is a very potent inducer of apoptosis because it inhibits many different kinases. Staurosporine-induced apoptosis has been discussed for various tumor therapies (168). The prolyl-hydroxylase inhibitor dimethyloxalylglycine activates the hypoxia-inducible factor (HIF)-1 pathway by stabilizing HIF-1α and has a protective effect against ischemia/reperfusion injury (169). Dimethyloxalylglycine may be protective against AKI. Sulpiride, an antipsychotic with selective dopaminergic antagonist properties, has a therapeutic effect in COVID-19 patients with psychiatric disorders (170, 171). With regard to diseases as risk factors of COVID-19 infection, such as cancer, other infections, and organ damage, the above drugs all have potential therapeutic effects. Further investigation is needed for confirmation.

## 5 Conclusion

Our study summarizes relationships among COVID-19, AKI, and CKD through bioinformatics analysis and identifies the potential molecular mechanism by which SARS-CoV-2 infection affects renal function. We examined 17 DEGs from three datasets by GO analysis and identified oxidative metabolism as the major biological function of these genes. Moreover, pathway enrichment analysis revealed that the MAPK signaling pathway, the IL-1 structural pathway, and the Toll-like receptor pathway, which are important pathways of systemic and organ inflammation pathology, are pivotal in the occurrence of AKI, CKD, and COVID-19. This study suggests that these pathways are involved in the mechanisms of AKI in COVID-19 patients and the deterioration of renal function. Then, the four most significant hub genes were screened from the PPI network and found to be closely related to the inflammatory response and tissue injury. In addition, the TFs and miRNAs identified play crucial roles in different functional disorders. Different types of diseases related to DEG mutations, mainly reproductive phylogenetic problems, psychophysiological disorders, and cancers, are shared complications of the three diseases. Analysis of COVID-19, AKI, and CKD provides a way to identify the pathogenesis of various diseases and helps in further understanding the underlying mechanisms of the development of AKI and the progression of CKD in COVID-19 patients. Therefore, it is possible to reduce the risk of SARS-CoV-2 infection resulting in AKI and CKD. However, COVID-19 is a newly discovered disease that has not been thoroughly studied, and more data are needed for further research. Multiomics analysis of COVID-19 is becoming important with the availability of bioinformatics approaches. Further cohort follow-up may help to elucidate the molecular mechanisms of AKI and CKD development in COVID-19 patients. This study provides promising pathways and molecular biomarkers for the association of COVID-19 with kidney diseases, and the findings are significant for the effective treatment of COVID-19.

## Data availability statement

The datasets presented in this study can be found in online repositories. The names of the repository/repositories and accession number(s) can be found in the article/**Supplementary Material**.

## Author contributions

QY and FL conceived and designed the research. WZ and LL wrote the paper. XX, HZ, ZP, WW, LH, YX, and LT revised the paper. HX, WN, and XY generated and provided analytical tools. WZ, LL, and XX analyzed data. All authors contributed to the article and approved the submitted version.

## Supplementary material

Supplementary Figure 1 Expression of 17 common DEGs in three datasets. Panels **(A–C)** represent the expression of common DEGs in GSE1563, GSE66494, and GSE147507, respectively. In the heatmap, each row represents the expression of a gene in different samples; red indicates that the gene is upregulated, and blue indicates that the gene is downregulated.

Supplementary Figure 2 Building COVID-19 diagnostic models and model interpretability **(A)** AUC of the 6 machine learning models in the training set. **(B)** AUC of the 6 machine learning models in the validation set. **(C)** Calibration plots were used to assess the agreement between predicted and observed values in different percentiles of predicted values. **(D)** Figure a is a SHAP diagram showing the relationship between each variable and the outcome. Each point represents a patient; the redder the color of the point indicates a larger value, and the bluer the color of the point indicates a smaller value. The farther to the right of the abscissa of the point, the greater the contribution to the predicted positive outcome, and the farther to the left of the abscissa of the point, the greater the contribution to the predicted negative outcome.

Supplementary Table 1 Results of molecular docking of drugs and proteins (kcal/mol).

Supplementary Table 2 Binding free energies and energy components predicted by MM/GBSA (kcal/mol).

Supplementary Table 3 Comparison of multiple models in training set.

Supplementary Table 4 Comparison of multiple models in validation set.
