# Supplementary figures and images for: Identification of common molecular signatures of SARS-CoV-2 infection and its influence on acute kidney injury and chronic kidney disease
Source: Front Immunol. 2023 Mar 21;14:961642. doi: 10.3389/fimmu.2023.961642 (PMC10070855; doi:10.3389/fimmu.2023.961642)

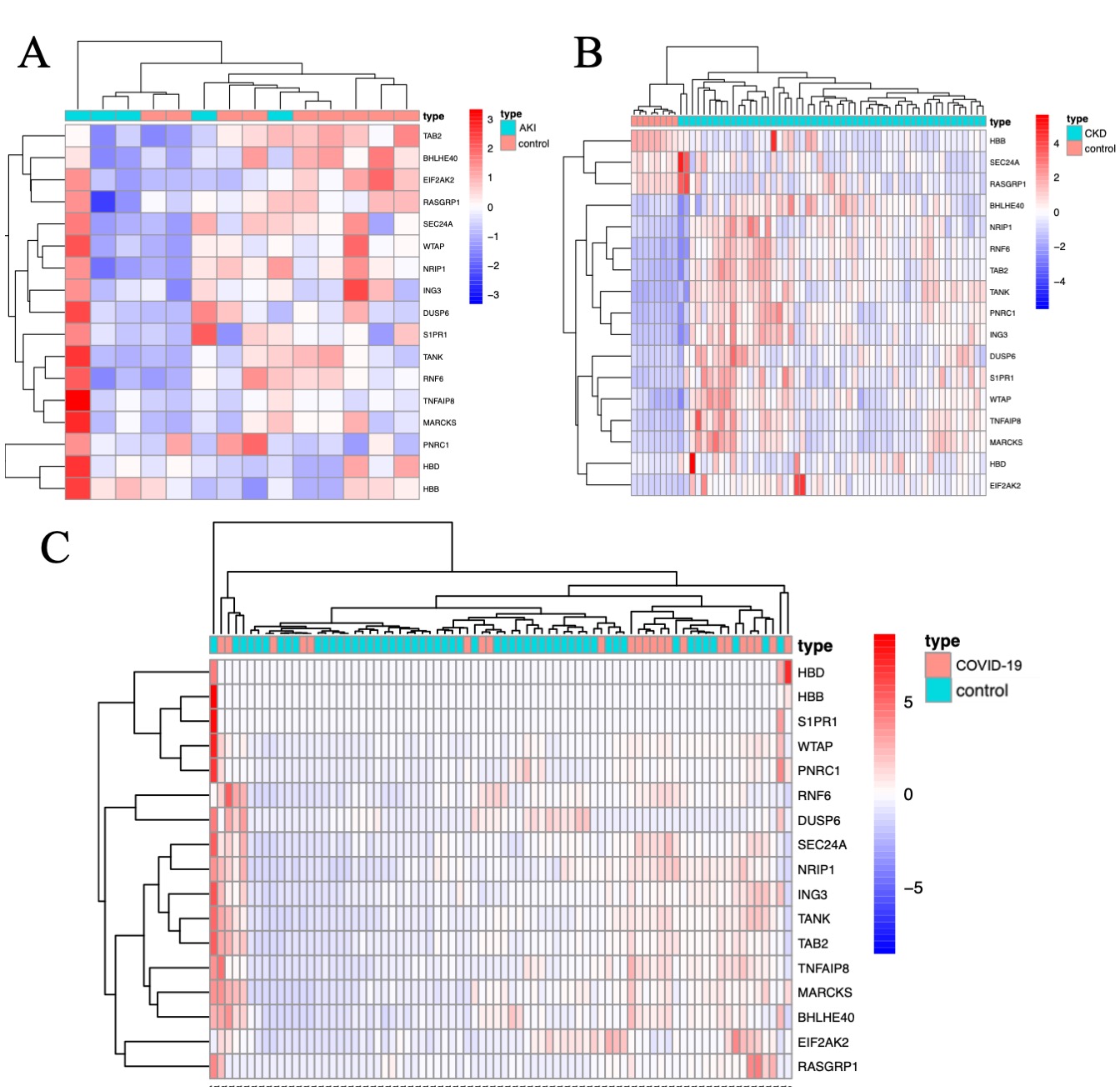

Supplement: Supplementary Figure 1 — Expression of 17 common DEGs in three datasets. Panels (A–C) represent the expression of common DEGs in GSE1563, GSE66494, and GSE147507, respectively. In the heatmap, each row represents the expression of a gene in different samples; red indicates that the gene is upregulated, and blue indicates that the gene is downregulated. [file Image_1.jpeg]

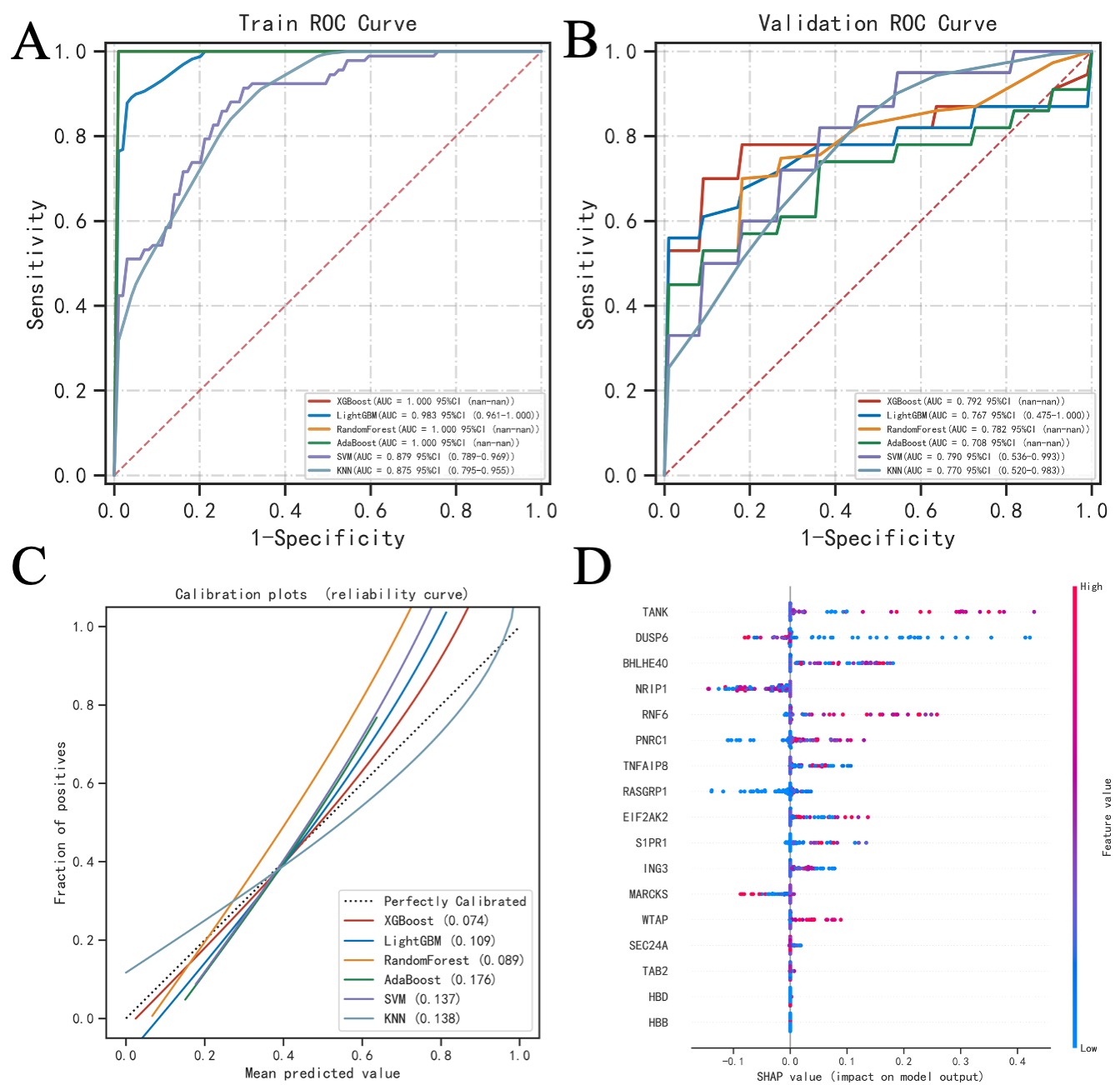

Supplement: Supplementary Figure 2 — Building COVID-19 diagnostic models and model interpretability (A) AUC of the 6 machine learning models in the training set. (B) AUC of the 6 machine learning models in the validation set. (C) Calibration plots were used to assess the agreement between predicted and observed values in different percentiles of predicted values. (D) Figure a is a SHAP diagram showing the relationship between each variable and the outcome. Each point represents a patient; the redder the color of the point indicates a larger value, and the bluer the color of the point indicates a smaller value. The farther to the right of the abscissa of the point, the greater the contribution to the predicted positive outcome, and the farther to the left of the abscissa of the point, the greater the contribution to the predicted negative outcome. [file Image_2.jpeg]
