# Supplementary material for: Identification of common molecular signatures of SARS-CoV-2 infection and its influence on acute kidney injury and chronic kidney disease
Source: Front Immunol. 2023 Mar 21;14:961642. doi: 10.3389/fimmu.2023.961642 (PMC10070855; doi:10.3389/fimmu.2023.961642)
Supplement: Supplementary Table 1 — Results of molecular docking of drugs and proteins (kcal/mol). [file Table_1.docx]

**SUPPLEMENTARY TABLE 1** Results of molecular docking of drugs and proteins (kcal/mol).

| **Drugs** | **DUSP6** | **BHLHE40** | **RASGRP1** | **TAB2** | **ACE2** | **3CLpro** |
| --- | --- | --- | --- | --- | --- | --- |
| **Tanespimycin** | -5.67 | -7.11 | -6.65 | -8.06 | -4.83 | -4.89 |
| **Camptothecin** | -5.41 | -6.68 | -7.12 | -8.43 | -6.38 | -5.59 |
| **Niclosamide** | -5.07 | -7.06 | -7.68 | -9.26 | -4.69 | -4.62 |
| **Pyrvinium** | -5.49 | -7.46 | -7.77 | -8.7 | -6.61 | -5.11 |
| **Staurosporine** | -5.0 | -6.09 | -6.01 | -6.81 | -5.92 | -4.86 |
| **Dimethyloxalylglycine** | -5.01 | -5.76 | -6.31 | -6.43 | -5.91 | -4.49 |
| **Sulpiride** | -4.54 | -4.39 | -5.47 | -6.63 | -5.77 | -4.21 |
| **Daunorubicin** | -5.09 | -5.19 | -5.34 | -8.21 | -5.39 | -5.21 |
