# Supplementary material for: Identification of common molecular signatures of SARS-CoV-2 infection and its influence on acute kidney injury and chronic kidney disease
Source: Front Immunol. 2023 Mar 21;14:961642. doi: 10.3389/fimmu.2023.961642 (PMC10070855; doi:10.3389/fimmu.2023.961642)
Supplement: Supplementary Table 2 — Binding free energies and energy components predicted by MM/GBSA (kcal/mol). [file Table_2.docx]

**SUPPLEMENTARY TABLE 2**  Binding free energies and energy components predicted by MM/GBSA (kcal/mol).

| **System** | **VDW** | **EEL** | **EGB** | **ESURF** | **DELTA-TOT** |
| --- | --- | --- | --- | --- | --- |
| **Tanespimycin**‒**DUSP6** | -25.9384±1.3264 | -4.3270±2.8365 | 17.4379±2.3231 | -2.9582±0.1577 | -15.7857±1.3991 |
| **Pyrvinium**‒**RASGRP1** | -40.5615±1.0072 | 30.2290±2.5312 | -12.9713±2.6457 | -4.3872±0.0808 | -27.6909±0.9977 |
| **Niclosamide**‒**TAB2** | -28.8323±0.7885 | 18.2513±2.9969 | -0.4606±3.1325 | -3.8156±0.0735 | -14.8572±0.5838 |
| **Pyrvinium**‒**BHLHE40** | -30.2846±1.5732 | 5.1340±3.8963 | 6.8888±3.4105 | -3.3247±0.0918 | -21.5866±0.9644 |
| **Pyrvinium**‒**ACE2** | -43.8287±1.5023 | -310.8792±1.3346 | 331.4498±1.3050 | -5.2459±0.1244 | -28.5042±1.4538 |
| **Camptothecin**‒**3CLpro** | -22.3129±1.9202 | -7.8007±1.6113 | 19.7333±2.2517 | -2.8357±0.1831 | -13.2160±1.4146 |

VDW: van der Waals energy; EEL: electrostatic energy; EGB: electrostatic contribution to solvation; ESURF: non-polar contribution to solvation; DELTA-TOT: binding free energy.
