# Supplementary material for: Identification of common molecular signatures of SARS-CoV-2 infection and its influence on acute kidney injury and chronic kidney disease
Source: Front Immunol. 2023 Mar 21;14:961642. doi: 10.3389/fimmu.2023.961642 (PMC10070855; doi:10.3389/fimmu.2023.961642)
Supplement: Supplementary Table 3 — Comparison of multiple models in training set. [file Table_3.docx]

**SUPPLEMENTARY TABLE 3** Comparison of multiple models in training set

| **Models** | **AUC**  **(SD)** | **Cutoff**  **(SD)** | **Accuracy**  **(SD)** | **Sensitivity**  **(SD)** | **Specificity**  **(SD)** | **Positive predictive value (SD)** | **Negative predictive value (SD)** | **F1 score**  **(SD)** |
| --- | --- | --- | --- | --- | --- | --- | --- | --- |
| **XGBoost** | 1.000  (0.000) | 0.683  (0.049) | 0.984  (0.000) | 1.000  (0.000) | 1.000  (0.000) | 1.000  (0.000) | 0.978  (0.000) | 1.000  (0.000) |
| **LightGBM** | 0.983  (0.008) | 0.438  (0.045) | 0.933  (0.022) | 0.924  (0.044) | 0.945  (0.065) | 0.929  (0.054) | 0.935  (0.013) | 0.925  (0.024) |
| **RandomForest** | 1.000  (0.000) | 0.570  (0.081) | 0.981  (0.006) | 1.000  (0.000) | 1.000  (0.000) | 1.000  (0.000) | 0.974  (0.009) | 1.000  (0.000) |
| **AdaBoost** | 1.000  (0.000) | 0.565  (0.017) | 0.984  (0.000) | 1.000  (0.000) | 1.000  (0.000) | 1.000  (0.000) | 0.978  (0.000) | 1.000  (0.000) |
| **SVM** | 0.879  (0.019) | 0.244  (0.036) | 0.779  (0.032) | 0.892  (0.088) | 0.755  (0.068) | 0.597  (0.067) | 0.921  (0.034) | 0.709  (0.031) |
| **KNN** | 0.875  (0.017) | 0.400  (0.000) | 0.804  (0.027) | 0.880  (0.043) | 0.705  (0.038) | 0.776  (0.059) | 0.810  (0.023) | 0.824  (0.047) |
