# Supplementary material for: Identification of common molecular signatures of SARS-CoV-2 infection and its influence on acute kidney injury and chronic kidney disease
Source: Front Immunol. 2023 Mar 21;14:961642. doi: 10.3389/fimmu.2023.961642 (PMC10070855; doi:10.3389/fimmu.2023.961642)
Supplement: Supplementary Table 4 — Comparison of multiple models in validation set. [file Table_4.docx]

**SUPPLEMENTARY TABLE 4** Comparison of multiple models in validation set

| **Models** | **AUC**  **(SD)** | **Cutoff**  **(SD)** | **Accuracy**  **(SD)** | **Sensitivity**  **(SD)** | **Specificity**  **(SD)** | **Positive predictive value (SD)** | **Negative predictive value (SD)** | **F1 score**  **(SD)** |
| --- | --- | --- | --- | --- | --- | --- | --- | --- |
| **XGBoost** | 0.792  (0.182) | 0.683  (0.049) | 0.807  (0.040) | 0.780  (0.204) | 0.891  (0.068) | 0.853  (0.181) | 0.814  (0.025) | 0.801  (0.172) |
| **LightGBM** | 0.767  (0.164) | 0.438  (0.045) | 0.832  (0.055) | 0.690  (0.174) | 0.909  (0.141) | 0.800  (0.187) | 0.841  (0.044) | 0.721  (0.118) |
| **RandomForest** | 0.782  (0.151) | 0.570  (0.081) | 0.758  (0.089) | 0.740  (0.174) | 0.855  (0.093) | 0.730  (0.248) | 0.781  (0.054) | 0.707  (0.174) |
| **AdaBoost** | 0.708  (0.192) | 0.565  (0.017) | 0.796  (0.044) | 0.690  (0.310) | 0.855  (0.212) | 1.000  (0.000) | 0.777  (0.042) | 0.772  (0.243) |
| **SVM** | 0.790  (0.094) | 0.244  (0.036) | 0.652  (0.104) | 0.870  (0.166) | 0.745  (0.121) | 0.460  (0.102) | 0.846  (0.048) | 0.597  (0.120) |
| **KNN** | 0.770  (0.087) | 0.400  (0.000) | 0.729  (0.070) | 0.830  (0.087) | 0.636  (0.163) | 0.603  (0.244) | 0.774  (0.029) | 0.660  (0.160) |
